# Motion characteristics of subclinical tremors in Parkinson’s disease and normal subjects

**DOI:** 10.1038/s41598-022-07957-z

**Published:** 2022-03-07

**Authors:** Ping Yi Chan, Zaidi Mohd Ripin, Sanihah Abdul Halim, Wan Nor Arifin, Ahmad Shukri Yahya, Gaik Bee Eow, Kenny Tan, Jyh Yung Hor, Chee Keong Wong

**Affiliations:** 1 School of Engineering, Monash University Malaysia, Jalan Lagoon Selatan, Bandar Sunway, 47500 Subang Jaya, Selangor Malaysia; 2 School of Mechanical Engineering, Universiti Sains Malaysia, Engineering Campus, Nibong Tebal, Penang Malaysia; 3 Department of Medicine, School of Medical Sciences, Universiti Sains Malaysia, Health Campus, Kubang Kerian, Kelantan Malaysia; 4 Biostatistics and Research Methodology Unit, School of Medical Sciences, Health Campus, Kubang Kerian, Kelantan Malaysia; 5 School of Civil Engineering, Universiti Sains Malaysia, Engineering Campus, Nibong Tebal, Penang Malaysia; 6 Department of Neurology, Penang General Hospital, Georgetown, Penang Malaysia

**Keywords:** Parkinson's disease, Statistics, Scientific data, Biomedical engineering, Parkinson's disease

## Abstract

The characteristics of the Parkinson’s disease tremor reported previously are not applicable to the full spectrum of severity. The characteristics of high- and low-amplitude tremors differ in signal regularity and frequency dispersion, a phenomenon that indicates characterisation should be studied separately based on the severity. The subclinical tremor of Parkinson’s disease is close to physiological tremor in terms of amplitude and frequency, and their distinctive features are still undetermined. We aimed to determine joint motion characteristics that are unique to subclinical Parkinson’s disease tremors. The tremors were characterised by four hand–arm motions based on displacement and peak frequencies. The rest and postural tremors of 63 patients with Parkinson’s disease and 62 normal subjects were measured with inertial sensors. The baseline was established from normal tremors, and the joint motions were compared within and between the two subject groups. Displacement analysis showed that pronation–supination and wrist abduction–adduction are the most and least predominant tremor motions for both Parkinson’s disease and normal tremors, respectively. However, the subclinical Parkinson’s disease tremor has significant greater amplitude and peak frequency in specific predominant motions compared with the normal tremor. The flexion–extension of normal postural tremor increases in frequency from the proximal to distal segment, a phenomenon that is explainable by mechanical oscillation. This characteristic is also observed in patients with Parkinson’s disease but with amplification in wrist and elbow joints. The contributed distinctive characteristics of subclinical tremors provide clues on the physiological manifestation that is a result of the neuromuscular mechanism of Parkinson’s disease.

## Introduction

Characteristics of Parkinson’s disease (PD) tremors in general have been studied extensively. The classical rest tremor, isolated postural tremor and kinetic tremor during slow movement have been reported as 3–7 Hz^1,2^, 4–9 Hz^2^ and 7–12 Hz^2^, respectively. The peak of rest tremor was found not only at fundamental frequencies but also at harmonics^1^. These characterisations were made on tremors that were visible and are not be applicable to the full spectrum of severity.

One of the few studies on PD of low and high amplitudes and normal subjects reported the tremor characteristics based on the signal regularity or predictability of the future value in time series. The regularity was quantified by approximate entropy, the amplitude of which indicates the randomness of a signal. Approximate entropy ranges from 0 to 2; the value 0 indicates accurate short- and long-term predictions of future value, as seen in a sine wave; the value 2 indicates that a signal is highly randomised, such as white Gaussian noise^3^. The researchers showed that the approximate entropy gradually decreases across the subject group from the control, least affected limb to the most affected limb of the patient with PD. In other words, low-amplitude tremor has a less regular signal.

Another parameter that has been studied in PD tremors of different severity is the proportion of acceleration power. It is a ratio of power in an individual frequency bin to the total power from 1 to 30 Hz in a power spectral analysis. Individual frequency bins are obtained by equally splitting the full frequency spectrum by 38 segments. When considering a patient with PD, the proportion of power at around 9 Hz in the least affected limb is less than in the most affected limb; this pattern is the opposite at 16–30 Hz^3^. The difference in the characteristics of high and low amplitudes suggests that PD tremors should be studied at a separate severity level.

A subclinical PD tremor or tremor that is not easily detectable in PD is known to be close to physiological tremor in terms of amplitude and frequency^3^. Based on previous studies, the amplitude of oscillation and/or the peak frequency alone is insufficient to establish a significant difference between the two types of tremors^3,4^. Researchers have analysed subclinical tremors and have not found evident accelerometric traces through signal bursts in electromyography^5,6^. To the best of our knowledge, only three studies have included the characteristics of subclinical tremor based on inertial measurement data and the data were compared with those of controls^4,7,8^. Beuter et al. found that subclinical PD tremors have a greater proportion of power at 4–6 Hz compared with controls^4^. This finding agrees with the frequency range that is normally found in a PD rest tremor. Although not clinically detectable, the similar characteristics of a typical visible PD tremor indicate that a subclinical tremor occurs when central oscillators are active^9^. The central oscillators are responsible for PD tremor and in other words, the oscillatory properties of the central neural networks trigger the tremor. This also suggests that the distribution of neuronal degeneration in the substantia nigra has the common influence on tremor whether the amplitude is visible or subclinical^4^. Researchers have also shown other time- and frequency-varying properties that are significantly different in subclinical tremors of patients with PD and controls^4,7^. Nevertheless, the distinctive feature of these two types of tremors are still unclear and identifying more characteristics from different aspects will help in disease amelioration.

There are also clinical impressions (observations made without thorough scientific study) of certain joint motions, particularly flexion–extension at the wrist, pronation–supination at the elbow and finger flexion (pill-rolling)^10^, but no relevant evidence from the measurement of the subclinical PD tremor is available. Quantifying tremors in joint motion and different segmental locations should provide more detailed phenomenological data on the unobservable tremors.

In this observational study, we aimed to identify the joint motion characteristics that are unique to patients with PD with no signs of clinical tremor. We hypothesised that the displacement and frequency of wrist and elbow motions differ in subclinical PD and normal tremors. The key clinical findings from the study are the predominance of certain joint motions and distinctive tremor motion characteristics of PD with subclinical tremor. Furthermore, physiological tremor has been used to explain the origin of pathological tremor^11^, which suggests the importance of tremors in normal subjects. Thus, we examined the tremor data from normal subjects and compared the tremor motion within and between normal subjects and patients with PD. Our findings provide the physiological presentation that may help explain the neuromuscular mechanism in further study.

## Results

### Clinical characteristics of the subjects

In this study, the median ages of the 63 patients with PD and 62 normal subjects who participated are 69 years (interquartile range [IQR] = 11.0) and 51 years (IQR = 15.8), respectively. The percentage of male subjects is 66.7% in the PD group and 50.0% in the control group. The median predicted ratings of patients for rest, outstretching and wing postures are 0.2 (IQR = 0.2), 0.2 (IQR = 0.2) and 0.2 (IQR = 0.2), respectively. For the normal tremor, the median predicted ratings for rest, outstretching and wing postures are 0.0 (IQR = 0.0), 0.0 (IQR = 0.0) and 0.0 (IQR = 0.1), respectively.

The durations from the last intake of medication to the first measurement differ among subjects. An estimated medication wear-off period of 3 h was used as a reference to characterise the recruited patients. Most subjects had taken the last dose of medicine ≥ 3 h before the measurements (n = 45, 71.4%). Five (7.9%) subjects who could not report that duration were categorised as unknown for that criterion, and one subject was not on medication. These subjects took levodopa or levodopa-containing medicine. The median of the duration since the last medication intake and time since the first diagnosis of the disease are 2.0 h (IQR = 0.8 h) and 4.0 years (IQR = 6.0 years) respectively. The median levodopa equivalent dose consumed by the patients in subgroup I (subgroup with medication intake within 3 h) is 100 mg (IQR = 100 mg). The levodopa equivalent dose was derived based on the standardized conversion scale and formulae reported by Tomlinson et al.^12^.

### Within-group tremor motion comparison

**Figure 1:**
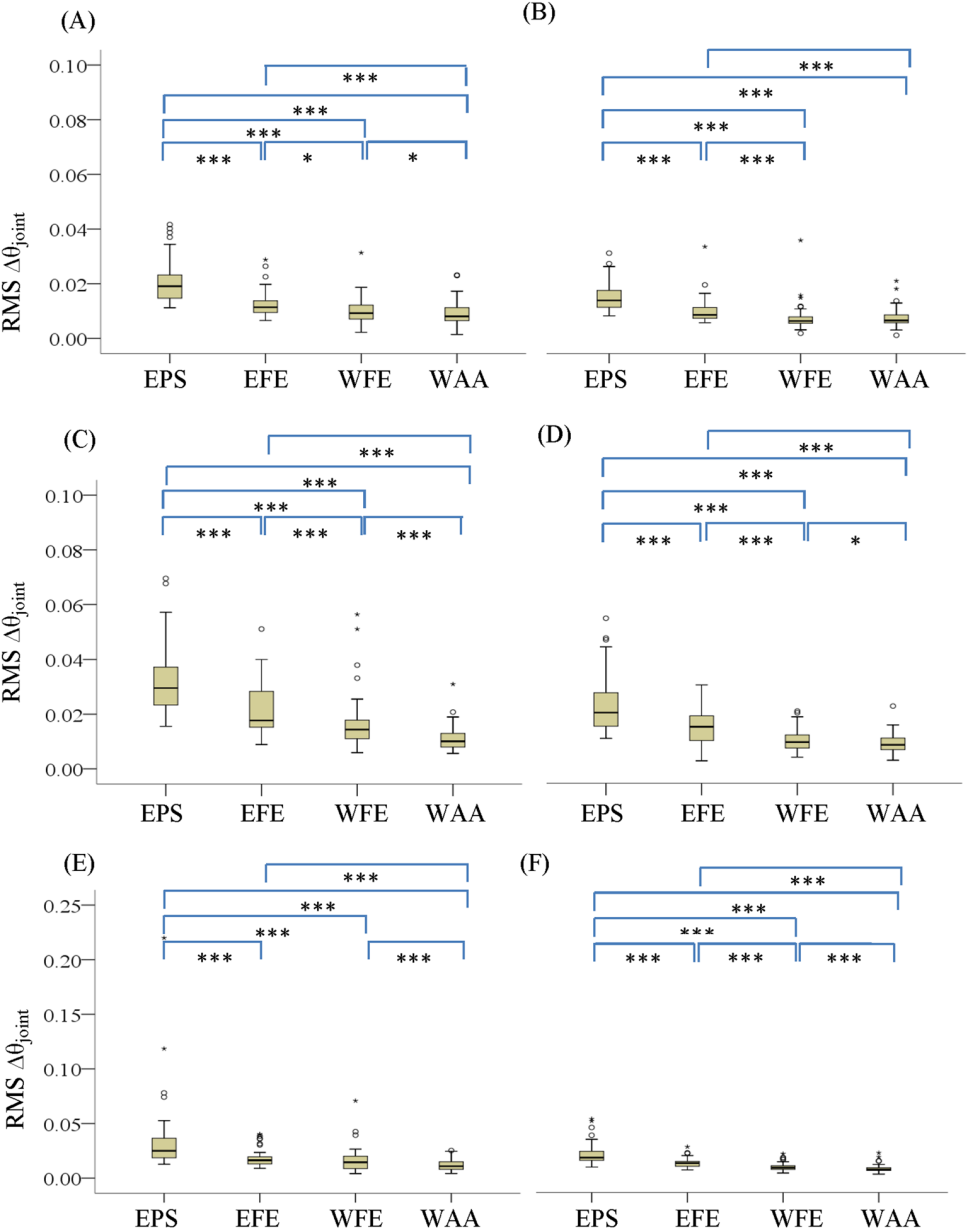
Boxplots of RMS $\Delta {\uptheta }_{{{\text{joint}}}}$ of subclinical tremors measured during resting (**A**,**B**), outstretched (**C**,**D**) and wing (**E**,**F**) postures for PD (**A**,**C**,**E**) and normal subjects (**B**,**D**,**F**). The significant difference is reported at *p < Bonferroni adjusted alpha levels, **p < 0.001 and ***p < 0.0001.

Different motions within each type of tremor were compared using the root mean square (RMS) of the joint angular displacement ($\Delta {\uptheta }_{{{\text{joint}}}}$) and peak frequency. Figure 1 shows the boxplots of the RMS $\Delta {\uptheta }_{{{\text{joint}}}}$ of subclinical PD tremors (Fig. 1A,C,E) and normal tremors (Fig. 1B,D,F) measured during resting, outstretching and wing postures. All the individual motions of the PD tremor are significantly different from one another in all postures except for the elbow flexion–extension (EFE) and wrist flexion–extension (WFE) in wing posture. The tremors of the controls have significant difference in all cases except for the tremor in WFE and wrist abduction–adduction (WAA) in the resting condition.

**Figure 2:**
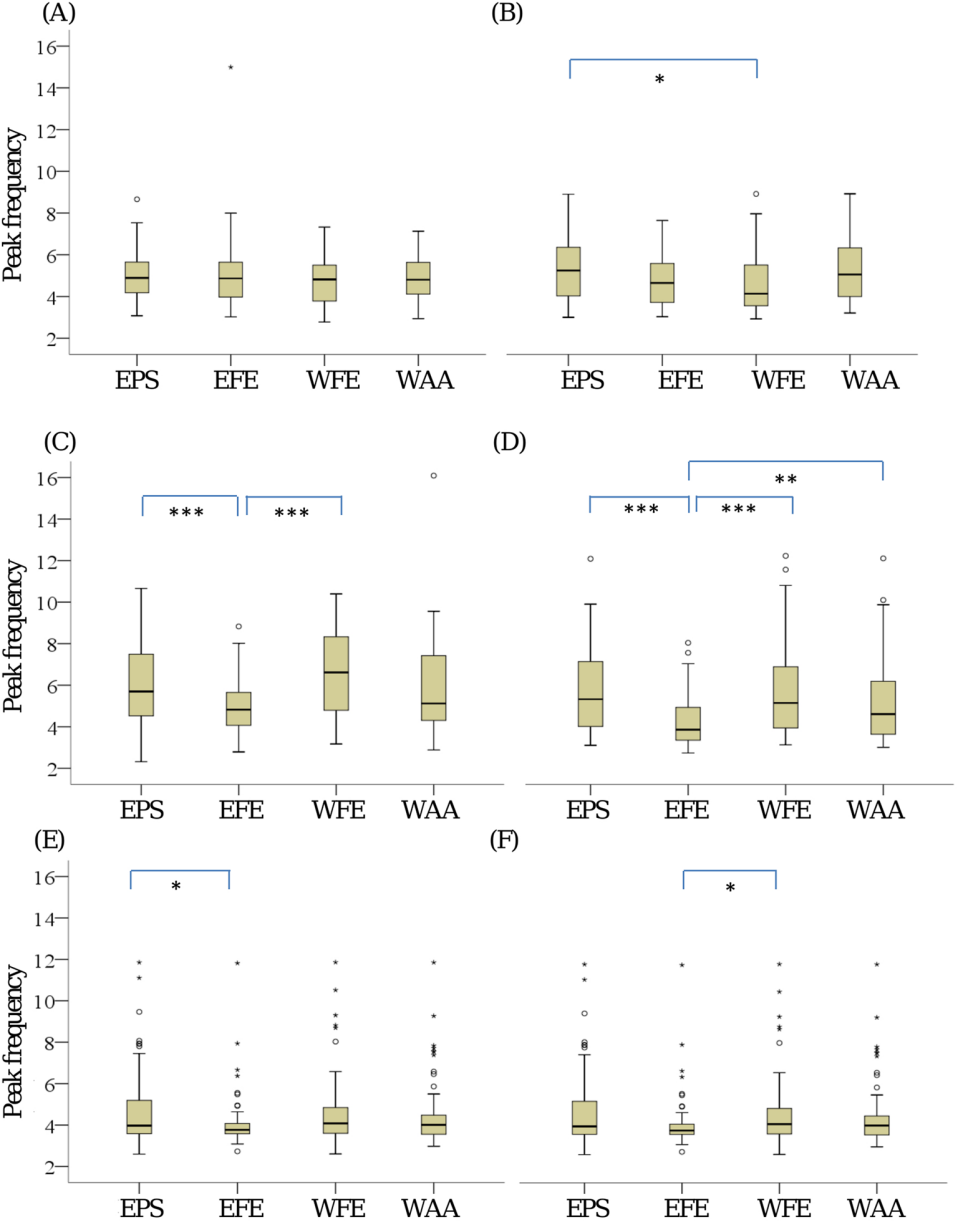
Boxplots of peak frequency of subclinical tremors measured during resting (**A**,**B**), outstretched (**C**,**D**) and wing (**E**,**F**) postures for PD (**A**,**C**,**E**) and normal subjects (**B**,**D**,**F**). The significant difference is reported at *p < Bonferroni adjusted alpha levels, **p < 0.001 and ***p < 0.0001.

The analysis using peak frequency as the tremor motion parameter in Fig. 2 shows that the values in individual tremor motions are not significantly different from each other except for the following cases of the PD tremor:

(i) Elbow pronation–supination (EPS) versus EFE and EFE versus WFE in outstretching posture;
(ii) EPS versus EFE in wing posture.

In the normal tremor, there are more significant differences:

(i) EPS versus WFE in the resting condition;
(ii) EFE versus any of three other tremor motions in outstretching posture;
(iii) EFE versus WFE in wing posture.

In all cases with a significant difference, the η^2^ (eta-squared) values are 0.09–0.77. These values indicate that the effect size of all the differences ranges from medium to large based on the interpretation guidelines provided by Cohen^13^: small effect, η^2^ = 0.01; medium effect, η^2^ = 0.06; large effect, η^2^ = 0.14.

### Between-group tremor motion comparison

**Figure 3:**
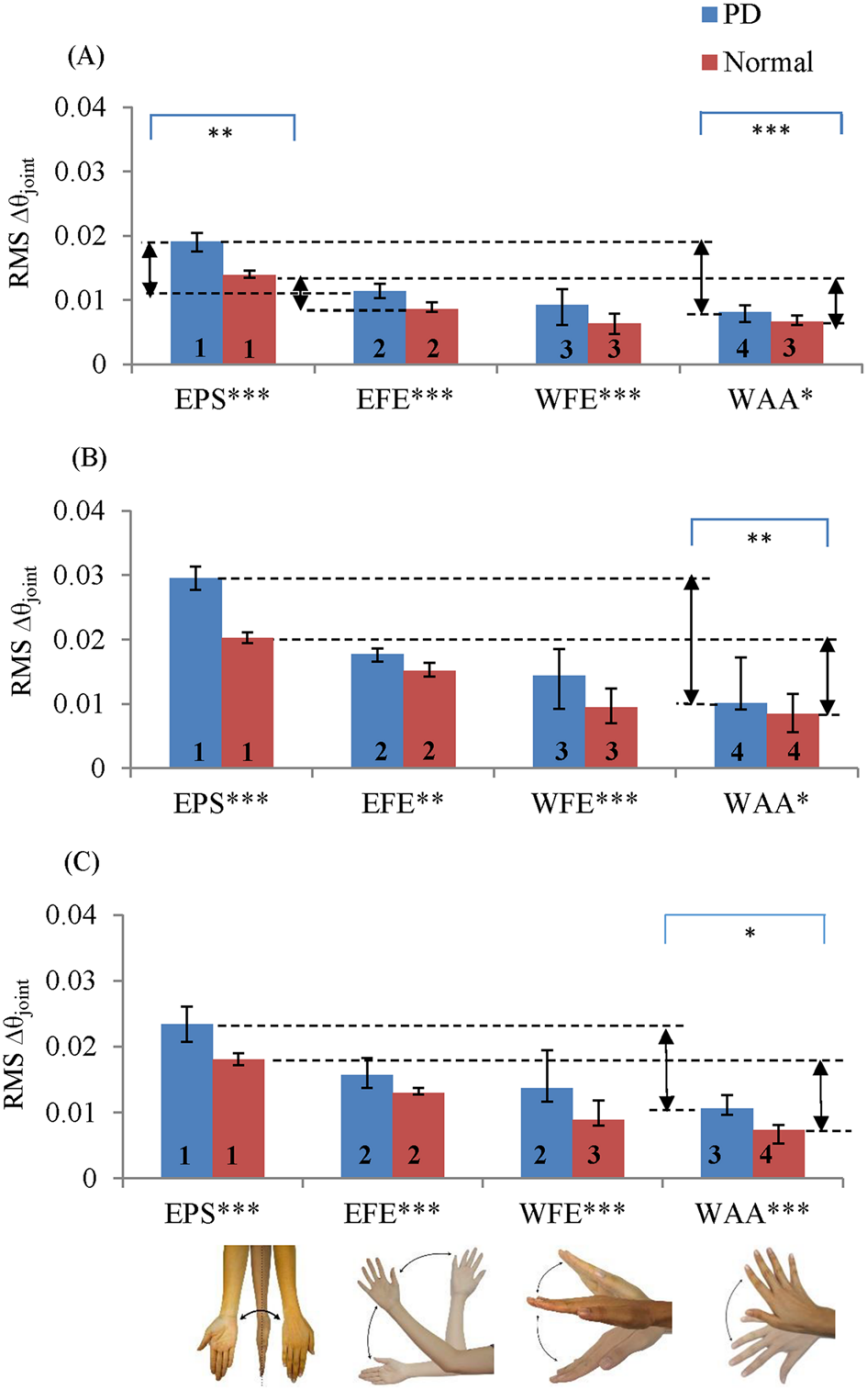
Median of RMS $\Delta {\uptheta }_{{{\text{joint}}}}$ of subclinical PD and normal tremors for (**A**) resting, (**B**) outstretching and (**C**) wing postures. The arrows indicate the relative severity of the pair motions. The severity rank is marked below each bar (higher rank indicates greater severity). The * marked above and below the graphs indicate the significant difference of the pair and individual motions respectively. The significant difference is reported at *p < 0.05, **p < 0.001 and ***p < 0.0001.

PD as well as normal tremors were rated < 0.5 based on the predicted rating. Nevertheless, the Kruskal–Wallis test using RMS $\Delta {\uptheta }_{{{\text{joint}}}}$ revealed that the PD tremor in every motion of three tested postures is significantly different from normal tremors. The median of RMS $\Delta {\uptheta }_{{{\text{joint}}}}$ values of the tremors is reported in Fig. 3A–C. The rankings of severity in the blue bars show that PD tremors have the following decreasing order of severity in rest and outstretching actions: EPS, EFE, WFE and WAA. In controls, the tremor severity ranks for all tremor motions are the same except for the wrist tremor motions in resting posture, which are not significantly different from each other. Thus, the WAA and WFE have the same rank (Fig. 3A).

Further analyses of the PD tremor showed that the median values of relative severity of EPS over EFE (EPS–EFE) and of EPS over WAA (EPS–WAA) are 1.75 and 1.5 times greater, respectively, than the median values of normal tremor during the resting condition (Fig. 3A; p < 0.001). Similarly, in outstretching and wing postures, the median values of the relative severity of EPS over WAA are 1.82 and 1.18 times greater, respectively, than those of the normal tremor (p < 0.005).

**Figure 4:**
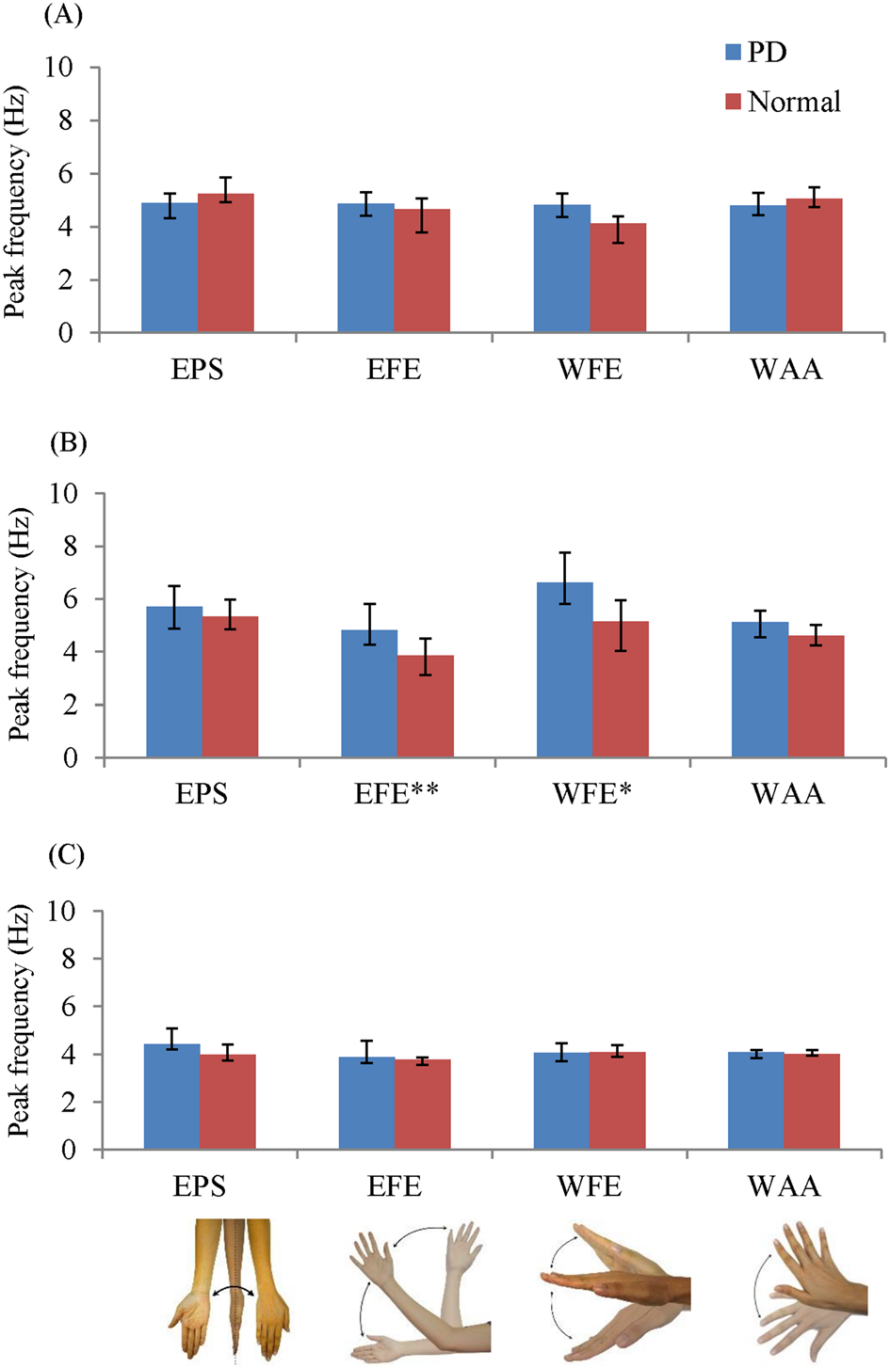
Median of peak frequency $\Delta {\uptheta }_{{{\text{joint}}}}$ of subclinical PD and normal tremors for (**A**) resting, (**B**) outstretching and (**C**) wing postures. The * marked below the graphs indicate the significant difference of the individual motions. The significant difference is reported at *p < 0.05, **p < 0.001 and ***p < 0.0001.

The analyses of peak frequency (Fig. 4) showed that the peak frequency of the PD tremor is close to that of the normal tremor in every joint motion of all actions, except in outstretching (Fig. 4B) posture, and the PD tremor has relatively higher frequency compared with the normal tremor for EFE and WFE. The peak frequency of the remaining cases ranges from 4.1 Hz (95% confidence interval [CI] 3.8, 4.8) to 5.7 Hz (95% CI 4.9, 6.8) in the PD tremor and 4.0 Hz (95% CI 3.8, 4.1) to 5.3 Hz (95% CI 4.2, 6.1) in the normal tremor.

The effects of most of the between-group comparisons with significant difference are medium to large based on the η^2^ of 0.06–0.22. The only cases with small effects are the between-group comparison based on EPS–WAA in terms of RMS $\Delta {\uptheta }_{{{\text{joint}}}}$ for wing action (η^2^ = 0.05) and WFE in terms of peak frequency for outstretching (η^2^ = 0.05). The values of amplitudes in RMS $\Delta {\uptheta }_{{{\text{joint}}}}$ and peak frequency and the corresponding η^2^ and p values are documented in the supplementary material (the sections ‘Baseline values and median of the PD tremor’, ‘Within-group comparisons’ and ‘Between-group comparison’).

### Important findings

The important findings in the study are summarised in Table 1. Especially in the between-group comparison, PD tremors are significantly different from normal tremors. The median values of the rest and postural conditions of baseline and PD tremors having no clinical sign based on predicted rating are reported for the first time.

**Table 1:** Summary of the tremor motion characteristics of subclinical PD and normal tremors.

Within-group comparison			
Parameter	Posture/action	Subclinical PD	Normal
RMS $\Delta {\uptheta }_{{{\text{joint}}}}$	Rest	WAA < WFE	No significant difference between WFE and WAA
	Wing	No significant difference between EFE and WFE	WFE < EFE
Peak frequency	Rest	No significant difference in all comparisons	WFE < EPS
	Outstretched	EFE < EPS and WFE	EFE < EPS, WFE and WAA
	Wing	EFE < EPS	EFE < WFE

PD tremor versus normal tremor			
Parameter	Posture/action	Motion-related parameters with significant difference	
RMS $\Delta {\uptheta }_{{{\text{joint}}}}$	Rest	EPS–EFE (pair-motion difference)	
	Rest, outstretched and wing	EPS–WAA (pair-motion difference)	
Peak frequency	Outstretched	EFE, WFE (individual motion)	

*RMS* $\Delta \theta_{joint}$ root mean square of the displacement about the joint, *WAA* wrist abduction–adduction, *WFE* wrist flexion–extension, *EPS* elbow pronation–supination, *EFE* elbow flexion–extension.

The severity ranking of the PD and normal tremors in increasing order is WAA, WFE, EFE and EPS. This order is found in all actions except for WFE and EFE in wing posture of PD tremors and the wrist motions in the resting condition of the normal tremor. The rank order indicates that in most of the tremor cases, the tremor about the elbow has a larger amplitude compared with the tremor about the wrist joint.

### Supplementary results

In addition, we studied the significant difference between the PD tremors in a subgroup with medication intake within 3 h (subgroup I), and tremors in another subgroup with medication intake within 3 h removed (subgroup II). The statistical analyses are tabulated in Supplementary Table 11. The result shows that the amplitudes of all individual tremor motions do not have significant difference in both groups. This indicates that the tremor amplitudes are similar for the different duration between the measurement and the last dose of medication intake (subgroup I, median = 2.0 h, IQR = 0.8 h; subgroup II, median = 5.0 h, IQR = 4.0 h).

When using pair-motion as tremor characteristics for comparison, consistent results were obtained. This finding is crucial to indicate the characteristic that the predominance of EPS compared to other motions is maintained even in patients taking medicine within 3 h.

A further analysis was carried out by removing PD with less than 3 h before comparing with the tremor of normal subject. The results are tabulated in Supplementary Table 12. The p-values show that EPS–EFE and EPS–WAA in resting, as well as EPS–WAA in outstretching are different in the patients and normal subjects. These characteristics are all found in the subjects with the medication intake of < 3 h included as well (refer to Supplementary Table 8). The difference when comparing the statistical results using tremors in subgroup II and all PD tremors is that the former does not result in EPS–WAA of wing posture that is unique from normal tremor. The p-values and η^2^ in all the cases show greater significant difference and effect sizes when all PD are included.

The boxplot of EPS–WAA in wing action of subgroup I (Supplementary Fig. 3) shows that it is relatively short and almost 100% of the data points have amplitudes that are within the range of tremor amplitude in subgroup II. This indicates that the predominance level of tremor in EPS over tremor in WAA is consistent in both subgroups. The median values of EPS–WAA during wing action in subgroup I (median 0.015°; IQR = 0.015°) and in subgroup II (median = 0.013°; IQR = 0.015°) are close. In overall, all the boxplots indicate that the two subgroups have similar amplitude of tremor in all the presented parameters. The median values of the individual and pair motion difference in subgroup II (Supplementary Tables 13 and 14 respectively) are available in the supplementary materials. The characteristics findings can be interpreted with the clinical characteristics of the subjects, particularly the median levodopa equivalent dose consumed by the patients in subgroup I.

## Discussion

### Tremor motion predominance and reason

Although the examined tremors had no clinical signs, our analysis revealed the characteristics that are unique to PD and normal tremors (Table 1). PD and normal tremors share the common characteristics of having EPS as the most predominant tremor motion and WAA as the least predominant tremor motion. Nevertheless, such predominance in the PD tremor is significantly greater than that of the normal tremor, as supported by a significantly larger EPS–WAA value in the former tremor. This finding suggests that the relative severity of the two motions in a PD tremor is not a mere manifestation of a physiological tremor.

A physiological tremor is chiefly a mechanical oscillation of which the frequency, $\omega$, is related to the inertia, *I*, and stiffness, *K*, in the Eq. (1)^14^.

1 $$\omega = \sqrt{\frac{K}{I}}$$

Researchers have reported that postural tremor in flexion–extension of the distal segment is greater than in the proximal segment^15,16^. This agrees with the flexion–extension of normal postural tremor in our study. We also found that it is distinctive in different motions for the same segments—for example, WAA has lower peak frequency than WFE. Another interesting finding is that the pathologic condition alters the tremor such that the peak frequencies are amplified in EFE and WFE only. In short, EFE and WFE in outstretching posture are the only motions that give a distinct difference between PD and normal tremors based on both amplitude and frequency. The findings of rank order and distinct peak frequency of the PD tremor in specific motion are new.

### Tremor motion characteristics found in other studies

To the best of our knowledge, no previous studies have included the characteristics of tremor motions of subclinical PD and normal tremors based on the readings of an inertial sensor. However, in two studies on the PD tremor, researchers showed that EPS is a dominant tremor motion. In one of these studies, the authors reported on the anecdotal impression that EPS is mainly observed in the PD tremor^17^, a phenomenon that agrees with our findings. Another clinical observational assessment on the tremors of 50 patients with PD further revealed that the rating in WAA is the lowest compared with WFE and EPS in outstretching posture (WAA rating = 0.05 ± 0.18; WFE rating = 0.17 ± 0.40; EPS rating = 0.15 ± 0.46) and wing posture (WAA rating = 0.01 ± 0.07; WFE rating = 0.17 ± 0.42; EPS rating = 0.12 ± 0.40)^18^. Nevertheless, the researchers did not perform statistical analysis to clarify any significant differences of the severity of the motions compared.

The previous findings comparing EPS versus WFE, however, do not exactly match the characteristics of PD found in our study. The reported relative severities of WFE and EPS differ depending on postures (WFE is most dominant in outstretching posture; EPS is most dominant in wing posture)^18^, contrary to our findings that tremor in EPS appears to be the most severe for all the tested actions. The possible reason for the different findings is the inclusion of visible tremor in the previous study. Nonetheless, further studies are required to understand the variation in the findings.

### PD tremor of low amplitude versus normal tremor of other studies

The research on comparing low-amplitude PD and normal physiological tremors is limited. The early comparison studies were done using RMS displacement, median frequency^4^ and peak frequency^3^, and on locations—namely, the finger^4^ and hand^3^. Although some patients with PD recruited in these studies had visible tremor^3,4^, the analyses showed that the two types of tremors are not distinguishable, particularly if only amplitude, of either displacement or frequency, is used for comparison. This highlights the strengths of our study: we identified distinctive features of the two tremor types that are all of rated < 0.5. The probable reason is that the system developed provides more degrees of freedom and more specific joint motions and locations for analysis. This leads to more comprehensive tremor characterisation even with only amplitude, rather than its derivatives. However, it is worth appreciating the studies that have investigated the transient characteristics^4^ and other derivatives^7^, because they have also provided other distinguishing features to the two tremor groups.

Previously, the frequencies of postural pathological tremor i.e. PD and essential tremor (ET) were generally reported to be indistinguishable from an enhanced physiological tremor^7^. Another study that showed no significant difference when comparing rest and postural tremors of controls and mild to moderate PD at peak power^3^ also supports that frequency is difficult for making the differentiation. Comparing the frequency at different tremor motions is new, and no previous clinical observation data are available for comparison.

### Generalisability, limitations and future work

Although the tremor quantification method is applicable to other joint motions, such as finger flexion, the Attitude and Heading Reference System (AHRS) of 5 cm × 3.6 cm × 2.5 cm (length, width and height) and 40 g may alter tremendously the dynamics of a finger. Hence, this makes the measurement system unsuitable for finger measurement. Although finger flexion was not included in the study, the analysis methodology is able to provide evidence in the tremor differentiation. Further work with a suitable sensing device could be done to quantify the importance of the finger flexion in a subclinical tremor. Moreover, we could also study the transient characteristics and derivatives such as power proportions to identify more characteristics that contribute to the tremor differentiation.

In order to understand if the subgroup with potential medication effects, i.e. subgroup I has any characteristic difference with the subgroup II, the two subgroups were compared and analysed. Based on the analyses, we found that the two subgroups do not differ significantly using either RMS $\Delta {\uptheta }_{{{\text{joint}}}}$ of the individual or pair motions.

When removing the subgroup I from the comparison between PD and normal subject tremors, most of the characteristics are retained in all actions except for the EPS–WAA in wing posture that the amplitudes have become no significant difference between subject groups. The significant difference based on the p-values of the rest of the parameters are found to drop with the removal of subgroup I as well.

There is a difference in the statistical findings of the comparison between normal and PD tremors when subgroup I is included and omitted. However, no clear evidence is found to show that the medicine altered any of the characteristics in subgroup I and the results of both boxplots and statistical analysis agree that no significant difference between subgroups I and II is noticed. In order to objectively report the findings, we provide the between-group comparison statistical results with or without the subgroup I in the results and supplementary material. The median values of the individual and pair motions after subgroup I is removed provided (refer to Supplementary Tables 13 and 14) can be interpreted with the levodopa equivalent dose and the duration since the last medication intake.

The motor-related side effects after prolonged intake of some of the tremor suppressing medications are motor fluctuation (‘on–off’ phenomenon) and dyskinesia^19^. The latter could potentially affect the measurements; hence, the patients with dyskinesia or any abnormal movement not related to tremor have been excluded from the study.

A question may arise whether a difference in the median age of the PD and normal subject groups affects the tremor characteristics. Normal healthy aging has been under the impression that it is a factor that alters physiological tremor^20^. Previously, clinical studies were carried out to investigate the effect of age on tremor. Sturman et al. compared the tremors of four age groups (young: n = 10, 20–30 years old; young-old: n = 10, 60–69 years old; old: n = 10, 70–79 years old; old-old: n = 10, 80–94 years old). They reported no significant difference in all four age groups based on the tremor amplitude. The oldest age group has lower modal frequency (peak frequency) and tremor regularity that is quantified with approximate entropy increases with age^21^. In another study that involved larger samples (59 young vs. 65 old healthy subjects), shows that no effect of age was found on the tremor frequency, acceleration amplitude and displacement amplitude^16^. Morrison et al*.* studied the tremor on 10 young healthy normotensive adults, 10 old, normotensive adults and 10 old, hypertensive adults. The results agreed that age alone does not reveal significant difference between all healthy young-old age group but the combined effects of age and cardiac disease have the greatest impact on the physiological tremor^20^. They speculate that measuring tremor on single body segment, as practised by previous studies^16,20,21^ may not provide a complete evaluation of tremor–age relation because tremor is usually observed throughout the limb^20^. In our opinion, until a systematic investigation is carried out explicitly, no solid conclusion can be drawn on the effect of age on tremor motion specifically.

The basic anthropometric information, particularly the weight and dimensions of the upper arm are useful to be related with the severity of tremor measured. Based on Eq. (1), the weight and upper limb dimensions, which contribute the inertia of the upper arm influences the frequency of the physiological tremor. Studying the mechanical oscillation in specific motion can be the future work.

The PD tremor amplitude is significantly greater than the normal tremor and particular tremor motions are unique to the PD tremor. Nevertheless, interpretation of clinical findings should be based on the reported characteristics of patients and measurement conditions. The clinical findings are applicable to tremors of the subject group with similar characteristics. Specifically, PD tremors have a rating < 0.5. The patients recruited are of Hoehn and Yahr stages I–IV, with 31% at stage I, 37% at stage II, 22% at stage III and 10% at stage IV. In a previous study, the disease stage did not correlate with the severity of tremor^22^ because the criteria of the stages are based mainly on the number of sides with symptoms, the ability to balance, mobility and other symptoms. This phenomenon explains the wide spectrum of disease stages among the patients with subclinical tremor.

### Clinical implication

Based on previous studies on the effect of ventrolateral thalamotomy on patients with PD, researchers have noted a reduction in tremor in the directions captured by sensors, particularly in EPS^23^. Based on our study, the subclinical PD tremor is not unidirectional. Rather, it is a symphony of joint motions that have unique predominance of amplitude and frequency in a specific direction. To the best of our knowledge, the neuromuscular mechanism that causes such combination of tremor motions has not yet been investigated. We hope our findings lead to more insights of the central oscillation and involuntary muscle actuation that is responsible for the subclinical PD tremor.

In conclusion, we have contributed evidence of the amplitude- and frequency-based predominance of tremor in individual motions and the joint locations at which a tremor is captured. Besides, the ability to draw a line between subclinical PD and control tremors based only on amplitudes suggests that joint motions provide a more comprehensive characterisation. Thus, characterisations with the derivatives, typically the temporal and frequency change in tremor motions, are worthy of further study. We have provided a clue to the physiological manifestation that is a result of neuromuscular mechanism of the subclinical PD tremor.

## Methods

### Study settings and participants

This is a cross-sectional study that centres around the characterisation of tremor based on hand–arm motion. The four hand–arm motions studied are WFE, WAA, EPS and EFE. There are two groups: patients with PD and controls (i.e. normal subjects). The study encompasses (i) the establishment of baseline values using the normal subject tremor readings, (ii) within-group tremor motion comparisons and (iii) between-group comparisons.

With the approval of the Medical Research Ethics Committee, Secretariat of National Institutes of Health, Malaysia, a 4-month study (protocol no. NMRR-14-1694-21740 [IIR]) was carried out mainly in the Neurology Clinic of Penang General Hospital. The research methods were carried out in accordance with the National Institutes of Health guidelines. Sixty-three patients with PD attending walk-in and appointment clinics and 62 normal subjects were recruited following written informed consent from normal and PD subjects or the legal guardians of the PD subjects. The total number of participants and missing data of each case are presented in the supplementary material (the section ‘Participation of the study subjects’).

All subjects were recruited based on a several inclusion criteria. All subjects were adults aged ≥ 40 years. The patients with PD had been diagnosed with idiopathic PD based on the United Kingdom Parkinson’s Disease Society Brain Bank clinical diagnostic criteria^24^ by neurologists and registrars in the Neurology Clinic. They were screened to include those with no significant clinical tremor by using a predicted tremor rating < 0.5 as one of the inclusion criteria. These tremor ratings were computed using a regression model that relates the average observational ratings from six doctors and the readings of the biomechanical system in a previous study^25^. The predicted rating in assessing tremor occurring during rest and outstretching postures is equivalent to the MDS-Unified Parkinson’s Disease Rating Scale (MDS-UPDRS), and the predicted rating in assessing tremor during wing posture is equivalent to the Washington Height-Inwood Genetic Study of Essential Tremor (WHIGET) rating scale (wTRS). A rating of 1 indicates the minimum tremor severity that is observable based on both the MDS-UPDRS and wTRS. Setting a predicted rating of 0.5 as a threshold is a more conservative means to categorise a tremor as a non-significant clinical sign. Other than screening the patients based on the tremor severity, the patients with dyskinesia were also excluded from the study.

The key criterion to include the normal subjects was the absence of tremor-related disease or illness. Specifically, the normal subjects do not have neurological disorders or illnesses that cause tremor. The exclusion criterion for both groups was the intake of substances or drugs that induce or suppress tremor, with the exception of the intake of PD medication for the patients with PD. The inclusion and exclusion criteria were designed based on the objective to find the characteristics of subclinical tremors.

### Procedure

Before the measurement, the tremor-related illness history, disease duration and time since last dose of tremor-suppressing medicine were recorded. Each subject was then asked to count numbers in decreasing order with two per interval and perform the resting, outstretching and wing postures for a specified time. The upper limb resting and outstretching postures were performed according to the protocols in the MDS-UPDRS upon attainment of permission from the International Parkinson and Movement Disorder Society, and the wing posture was done according to the protocol of the wTRS^26^. The following actions were performed. We provided the images of the postures in the supplementary material (section ‘Tremor measurement and postures’):

(i) The upper limb was rested on the arm rest for 15 s.
(ii) The upper limb was outstretched in front of the chest (with palm facing down) for 15 s.
(iii) The arm was held in the wing position (fully flexed at elbow and the index finger points towards thorax) for 15 s.

Because the patients were not recruited upon appointment, some of them took tremor-suppressing medicine, and there were different durations between the measurement and the last dose of medication (refer to the ‘Clinical characteristics of the subjects’ section in the ‘Results’ for the data).

### Tremor measurement

Four hand–arm motions were quantified with the use of an AHRS. An SBG IG-500A AHRS (SBG Systems, Rueil-Malmaison, France) consists of triaxial magnetometers, triaxial accelerometers and triaxial gyroscopes. The accelerometers and gyroscopes were sampled at 10 kHz. After a built-in Kalman filtering within the AHRS, the output data frequency was 100 Hz.

To measure the relative motion of the wrist and elbow joints, one AHRS was affixed on the hand, the lower arm and the upper arm. The quaternion in each AHRS was processed to determine the joint angle^25^. Subsequently, a fourth-order Butterworth bandpass filtering with a passband of 3–30 Hz was performed to limit the signals to contain only physiological and pathological tremors, which were previously found to be within this range^27–29^. The resulting parameter of the filtering is termed $\Delta {\uptheta }_{{{\text{joint}}}}$, which is essentially the displacement about the joint (in terms of degree, °) that includes mainly tremulous information. The discrete values of $\Delta {\uptheta }_{{{\text{joint}}}}$ in the entire measurement duration were used to compute the RMS of $\Delta {\uptheta }_{{{\text{joint}}}}$. The serial data were also subjected to fast Fourier transform spectral computation using a Hanning window. The process of computing the RMS and frequency spectrum were applied to the $\Delta {\uptheta }_{{{\text{joint}}}}$ in four tremor motion—that is, EFE, EPS, WAA and WFE.

The RMS and peak frequency of $\Delta {\uptheta }_{{{\text{joint}}}}$ were the key characterisation parameters of the tremor motions. The measurements were performed by research assistants, and the data acquisition and data processing from computing the joint angle until the attainment of RMS and peak frequency of $\Delta {\uptheta }_{{{\text{joint}}}}$ were done automatically in the LabVIEW software (National Instruments Corporation, Austin, Texas).

The measurement system was validated by comparing the RMS of angular displacement of the system and the rotary encoder system within a tremor simulator in the laboratory. The same AHRS were mounted on the tremor simulator that was actuated by servomotors. The coefficient of determination—R^2^ of the linear regression relating the RMS of angular displacement of the two systems—is 1.0000 (p < 0.001)^25^. In measuring PD tremors during resting, outstretching and wing actions for clinical validation, we previously found with regression analysis that the reading of the measurement system can explain more than 80% of the variability of the doctor’s observational rating (R^2^ > 0.80)^25^.

### Statistical analysis

Non-parametric statistics were used for the analyses because the data were not normally distributed. The analyses were carried out using SPSS Statistics for Windows version 23.0 (IBM Corp., Armonk, NY, USA). For within-group comparisons, the Wilcoxon signed rank test was used to test the statistical significance of the difference between the readings in four tremor motions. For comparisons that yielded a significant difference, the severity of the motions was ranked according to the magnitude of the parameter analysed. When performing multiple comparisons, some statistical tests may result in p values < 0.05 by chance; Holm’s sequential Bonferroni correction is one way to resolve the problem by adjusting the p values. Six sets of within-group comparisons are possible. The first to sixth most significant p values must be less than 0.008, 0.010, 0.013, 0.017, 0.025 and 0.050, respectively, to be considered to have a significant difference. The method to obtain all the significant p values is elaborated in the supplementary material (the section ‘Holm’s sequential Bonferroni correction’).

The Kruskal–Wallis test was used to compare the tremors between the normal and PD groups. The tremor characteristics compared between the two groups are the readings of individual tremor motions and the pair-motion difference (i.e. the difference between the most severe and other individual motions). The need to compare the pair-motion differences is based on a previous study^18^ that revealed the severity difference between EPS and WFE is unique in the PD tremor when quantified using an observational rating. We further examined the difference in all pair-motions in the hand and arm. The three pair-motion differences identified were the EPS–EFE, EPS–WFE and EPS–WAA after understanding that EPS is the most dominant motion. The effect size (η^2^) was calculated to evaluate the size of the difference of the tremor characteristics within the same subject group and between the two groups.

The 95% CI of the median of all parameters was estimated by the non-parametric bootstrap method because the data were not normally distributed^30^. In this method, 10,000 bootstrap samples were generated from the readings of each case of measurement (i.e. in resting and posture-maintaining conditions). Each bootstrap sample has the same sample size as the original sample, and all the tremor parameters (peak frequency and RMS of $\Delta {\uptheta }_{{{\text{joint}}}}$ in all motions) were associated with the same sets of subjects. The 10,000 median values were then computed.

### Supplementary study

A supplementary analysis was carried out to study if the inclusion of less than 3 h of medication intake before the measurement affects the overall tremor results. Statistical analysis was performed to compare the difference between PD with medication intake of within 3 h and another subgroup without medication intake of within 3 h. Another statistical analysis was done to compare the PD tremor with < 3 h medication intake removed, with the tremors of normal subjects. Kruskal–Wallis tests were performed for both statistical analyses.

## Supplementary Information

Supplementary Information.

## Data Availability

The dataset used and/or analysed during the current study available from the corresponding author on reasonable request.
