# Supplementary material for: Motion characteristics of subclinical tremors in Parkinson’s disease and normal subjects
Source: Sci Rep. 2022 Mar 7;12:4021. doi: 10.1038/s41598-022-07957-z (PMC8901710; doi:10.1038/s41598-022-07957-z)
Supplement: Supplementary file 1 — Supplementary Information. [file 41598_2022_7957_MOESM1_ESM.pdf]

Supplementary material

Motion Characteristics of Subclinical Tremors in Parkinson's Disease and Normal Subjects

Ping Yi Chan, PhD,[1*] Zaidi Mohd Ripin, PhD,[2] Sanihah Abdul Halim, MD, MMed,[3] Wan Nor Arifin, MBBS, MSc,[4] Ahmad Shukri Yahya, MSc,[5] Gaik Bee Eow, MD, FRCP,[6] Kenny Tan, MD, MRCP,[6] Jyh Yung Hor, MD, MRCP,[6] Chee Keong Wong, MBBS, MRCP[6]

1 School of Engineering, Monash University Malaysia, Bandar Sunway, Subang Jaya, Selangor, Malaysia

2 School of Mechanical Engineering, Universiti Sains Malaysia, Engineering Campus, Nibong Tebal, Penang, Malaysia

3 Department of Medicine, School of Medical Sciences, Universiti Sains Malaysia, Health Campus, Kubang Kerian, Kelantan, Malaysia

4 Biostatistics and Research Methodology Unit, School of Medical Sciences, Health Campus, Kubang Kerian, Kelantan, Malaysia

5 School of Civil Engineering, Universiti Sains Malaysia, Engineering Campus, Nibong Tebal, Penang, Malaysia

6 Department of Neurology, Penang General Hospital, Georgetown, Penang, Malaysia

Corresponding author
Dr Ping Yi Chan, Lecturer in School of Engineering, Monash University Malaysia, Jalan Lagoon Selatan, Bandar Sunway, 47500 Subang Jaya, Selangor, Malaysia; chan.pingyi@monash.edu

1. Tremor measurement and postures

Supplementary Figure 1 shows the three postures when the measurements of tremor were carried out. Mounted on the hand, lower arm and upper arm are the attitude and heading reference systems.

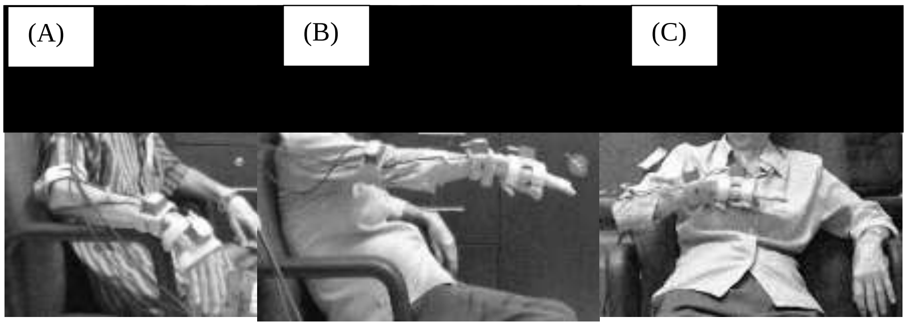

Supplementary Figure 1. (A) Rest, (B) outstretching and (C) wing postures when the tremors of subjects were measured.

2. Participation of the study subjects

Based on the previous study, the Parkinson's disease (PD) can occur as early as age 30–39 years [1] and the incidence rates for the 40–49-year age group for females and males are 3.26 and 3.57 per 100,000 person-years [2]. Therefore, healthy subjects in the 40–80-year age range were appropriate to be the controls for the study. Referring to Supplementary Figure 1, 82 patients recruited in previous study were assessed for eligibility. One patient was with dyskinesia and 18 more patients with predicted rating of more than 0.50 were excluded from the study. The reason for excluding patients with dyskinesia is that the measurement system has not tested under the condition with dyskinesia. The data of a total of 63 patients were available for the study. Apart from this, 62 normal subjects recruited in previous study were all included in this study.

Since some of the subjects did not perform all the rest, outstretching and wing actions, less than 63 sets of data per action are available. The number of missing of data is as tabulated in Supplementary Table 1. Manipulation was not done to replace the missing data.

3. Clinical characteristics of the subjects

The characteristics of the subjects involved in the study are reported in Supplementary Table 2. The median ages of the 63 PD and 62 normal subjects participated are 69 (interquartile range, IQR = 11.0) and 51 (IQR = 15.8), respectively. The percentage values of the male subjects in PD and control groups are 66.7% and 50.0% respectively. The durations from the last intake of medication to the first measurement differ among subjects. An estimated medication wear-off period of three hours was used as a reference to characterize the patients recruited. More subjects took the medicine for three or more hours (n = 45; 71.4%). Five (7.9%) subjects who could not report that duration were categorized as unknown for that criterion, and one subject was not on medication.

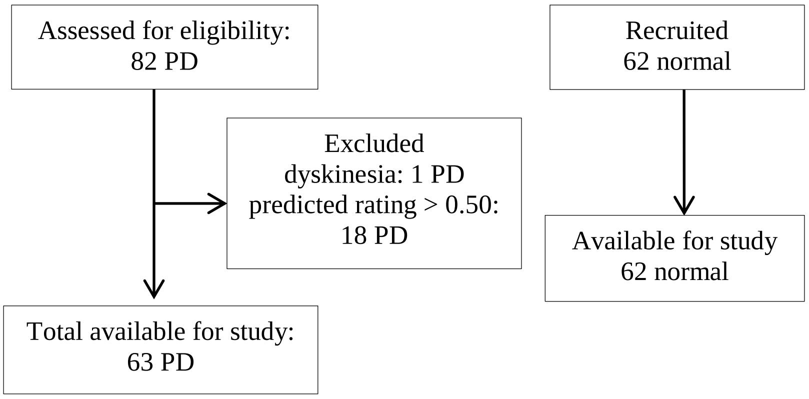

Supplementary Figure 2. Participation of PD patients (left) and normal subjects (right).

Supplementary Table 1. Number of available and missing data for the study.

Subject		Rest	Outstretching	Wing	Total
PD	Data set available	54	46	49	149
	Data with predicted rating > 0.50	28	33	21	
	Data set missing/ dyskinesia	0	3	12	
	Total	82	82	82	
Normal	Data set available	62	62	62	186
	Data set missing	0	0	0	
	Total	62	62	62	

Supplementary Table 2. Clinical characteristics of all the subjects.

		PD	Normal
		Resting, outstretching, wing (n = 63)	Resting, outstretching, wing (n = 62)
Median age, year (IQR; range)		69 (11.0; 52–86)	51 (15.8; 40–80)
Male, number (%)		42 (66.7)	31 (50.0)
Duration since last medication intake, number of patient (%)	<3 hours	13 (20.6)	–
	≥3 hours	44 (69.8)	–
	unknown	5 (7.9)	–
No medication intake, number of patient (%)		1 (1.6)	

Median of the duration since the last medication intake, hour (IQR)	<3 hours	2.0 (0.8)	-
	≥3 hours	5.0 (4.0)	-
Median number of years since the diagnosis of the disease (IQR)		4.0 (6.0)	
Median levodopa equivalent dose*consumed by the patients taking medicine within 3h, mg (IQR)		100 (100)	

* The levodopa equivalent dose is reported based on the standardized conversion scale and formulae reported by Tomlinson *et al.*[3].

4. Holm's sequential Bonferroni correction

When performing multiple comparisons, some statistical tests may result in p values of less than 0.05 by chance, so Holm's sequential Bonferroni correction is one way to resolve the problem by adjusting the p values. Six sets of within-group comparisons are possible. The first to sixth most significant p values must be less than 0.008, 0.010, 0.013, 0.017, 0.025, and 0.050, respectively, to be considered to have significant difference.

The adjusted p values used sequentially for the n pair of comparisons are:

$$\frac{\alpha}{n}, \frac{\alpha}{n-1}, \frac{\alpha}{n-2}, \ldots\ldots, \frac{\alpha}{1}$$

Where α is the alpha level.

In this study, six comparisons, namely EFE vs EPS, EPS vs WFE, EPS vs WAA, WAA vs WFE, EFE vs WFE and EFE vs WAA to evaluate the statistical significant difference between the readings in each comparison pair were performed. By using α of 0.05, the first to sixth most significant p values has to be less than 0.008, 0.010, 0.013, 0.017, 0.025 and 0.050 respectively to be considered to have significant difference.

5. Effect size

Variability with statistical significance may not reflect the practical importance. The effect size is a means for assessing the practical importance of an effect. For the within- and between-group comparisons, the effect is the difference between the compared parameters.

Based on the guidelines provided by Cohen, the following interpretation of the effect size can be made: small effect $\eta^2 = 0.01$; medium effect $\eta^2 = 0.06$; large effect $\eta^2 = 0.14$.[4]

6. Estimation of sample size based on previous literature

Since no previous study pertaining to the comparison of tremor motion among PD and normal subjects, the sample size of previous study was used as the reference. A clinical work published by Sternberg *et al.* reported the difference in the tremors of PD and essential tremor by observing the motions flexion-extension, abduction-adduction and pronation-supination of upper limb[5]. Fifty PD and 50 ET patients were determined as the sufficient number of subjects for the data to be statistically valid. The number of subject is determined based on the pre-study sample size calculation to achieve more than 90% confidence for the comparison. Since our work involves subject group comparison in tremor motion as well, 50 subjects per subject group was taken the approximated sample size in the clinical study.

7. Baseline values and median of PD tremor

Supplementary Table 3 shows the median of the PD and normal tremors. The baseline values of amplitudes in RMS $\Delta\theta_{joint}$ and peak frequency were established upon attaining significant difference between PD and normal tremors. The baseline RMS $\Delta\theta_{joint}$ values range from 0.006˚ (95% CI = 0.006˚, 0.007˚) to 0.014˚ (95% CI = 0.012 ˚, 0.016˚) in resting, 0.008˚ (95% CI = 0.008˚, 0.020˚) to 0.020˚(95% CI = 0.017˚, 0.023˚) in outstretching and 0.007˚ (95% CI = 0.007˚, 0.008˚) to 0.018˚(95% CI = 0.017˚, 0.021˚). The baseline peak frequency values range from 3.8Hz (95% CI = 3.7Hz, 3.9Hz) to 5.3Hz (4.2Hz, 6.1Hz). The median of relative tremor severity between pair motions of the control and PD tremors are tabulated in Supplementary Table 4.

Supplementary Table 3. Median of the tremor of PD (score < 0.5) and normal subjects.

	Action	Normal				Action	PD			
		EPS	EFE	WFE	WAA		EPS	EFE	WFE	WAA
$RMS\Delta\theta_{joint}$	Resting (n = 62)	0.014 (0.012, 0.015)	0.009 (0.008, 0.010)	0.006 (0.006, 0.007)	0.007 (0.006, 0.008)	Resting (n = 54)	0.019 (0.016, 0.022)	0.011 (0.010, 0.013)	0.009 (0.008, 0.011)	0.008 (0.007,0.009)
	Outstretching (n = 62)	0.020 (0.018, 0.023)	0.015 (0.012, 0.018)	0.009 (0.009, 0.010)	0.008 (0.008, 0.010)	Outstretching (n = 46)	0.030 (0.024, 0.034)	0.018 (0.017, 0.025)	0.014 (0.013, 0.016)	0.010 (0.009, 0.011)
	Wing (n = 62)	0.018 (0.017, 0.021)	0.013 (0.011, 0.014)	0.009 (0.008, 0.010)	0.007 (0.007, 0.008)	Wing (n = 49)	0.023 (0.021, 0.029)	0.016 (0.015, 0.018)	0.014 (0.011, 0.016)	0.011 (0.009, 0.013)
Peak frequency	Resting (n = 62)	5.2 (4.5, 5.5)	4.6 (4.3, 5.1)	4.1 (3.8, 4.7)	5.1 (4.2, 5.5)	Resting (n = 54)	4.9 (4.4, 5.3)	4.9 (4.5, 5.3)	4.8 (4.3, 5.2)	4.8 (4.4, 5.2)
	Outstretching (n = 62)	5.3 (4.2, 6.1)	3.9 (3.5, 4.3)	5.1 (4.7, 5.8)	4.6 (3.9, 5.3)	Outstretching (n = 46)	5.7 (4.9, 6.8)	4.8 (4.2, 5.3)	6.6 (5.8, 7.4)	5.1 (4.6, 6.1)
	Wing (n = 62)	4.0 (3.8, 4.3)	3.8 (3.7, 3.9)	4.1 (3.8, 4.5)	4.0 (3.8, 4.1)	Wing (n = 49)	4.4 (4.1, 4.8)	3.9 (3.6, 4.0)	4.1 (3.8, 4.7)	4.1 (3.8, 4.8)

The median values are in ˚. n = number of sample.

Supplementary Table 4. Median of the relative tremor severity between pair motions of PD (score < 0.5) and normal subjects.

Parameter	Action	Normal	Action	PD

		EPS – EFE	EPS – WFE	EPS –WAA		EPS – EFE	EPS –WFE	EPS – WAA
RMS$\Delta\theta_{joint}$	Resting (n = 62)	0.004 (0.004, 0.005)	0.007 (0.006, 0.008)	0.006 (0.005, 0.008)	Resting (n = 54)	0.007 (0.006, 0.009)	0.010 (0.007, 0.010)	0.009 (0.009, 0.012)
	Outstretching (n = 62)	0.007, (0.004, 0.007)	0.011 (0.008, 0.013)	0.011 (0.009, 0.014)	Outstretching (n = 46)	0.008 (0.005, 0.014)	0.014 (0.011, 0.019)	0.020 (0.015, 0.022)
	Wing (n = 62)	0.007 (0.004, 0.008)	0.010 (0.008, 0.011)	0.011 (0.009, 0.013)	Wing (n = 49)	0.008 (0.006, 0.011)	0.012 (0.010, 0.015)	0.013 (0.012, 0.019)

The median values are in ˚. n = number of sample.

8. Within-group comparison

The statistical results for within-group comparison using RMS $\Delta\theta_{joint}$ and peak frequency are tabulated in Supplementary Table 5 and 6 respectively. The explanation and discussions of the results are presented in the main manuscript.

a) RMS $\Delta\theta_{joint}$ of four tremor motions

Supplementary Table 5. Within-group comparison based on RMS $\Delta\theta_{joint}$.

Subject type	Action		EFE vs EPS	EPS vs WFE	EPS vs WAA	WAA vs WFE	EFE vs WFE	EFE vs WAA
PD (score < 0.50)	Resting (n = 54)	Z	-6.3	-6.1	-6.4	-2.2	-3.3	-4.9
		p	**<0.0001*** **	**<0.0001*** **	**<0.0001*** **	**0.025***	**0.001***	**<0.0001*** **
		η^2	0.74	0.70	0.77	0.09	0.20	0.46
	Outstretching (n = 46)	Z	-5.2	-5.1	-5.9	-4.8	-4.0	-5.8
		p	**<0.0001*** **	**<0.0001*** **	**<0.0001*** **	**<0.0001*** **	**<0.0001*** **	**<0.0001*** **

		η^2	0.60	0.57	0.76	0.51	0.35	0.75
	Wing (n = 49)	Z	-5.5	-5.6	-6.1	-4.3	-1.9	-5.3
		p	**<0.0001*** **	**<0.0001*** **	**<0.0001*** **	**<0.0001*** **	**0.058**	**<0.0001*** **
		η^2	0.64	0.66	0.77	0.38	0.07	0.59
Normal	Resting (n = 62)	Z	-6.48	-6.54	-6.83	-1.00	-5.28	-4.55
		p	**< 0.0001*** **	**< 0.0001*** **	**< 0.0001*** **	0.32	**< 0.0001*** **	**< 0.0001*** **
		η^2	0.69	0.70	0.77	0.02	0.46	0.34
	Outstretching (n = 62)	Z	-6.17	-6.84	-6.85	-3.25	-5.84	-6.24
		p	**< 0.0001*** **	**< 0.0001*** **	**< 0.0001*** **	**0.001***	**< 0.0001*** **	**< 0.0001*** **
		η^2	0.62	0.77	0.77	0.17	0.56	0.64
	Wing (n = 62)	Z	-6.8	-6.8	-6.8	-4.2	-5.6	-6.4
		p	**< 0.0001*** **	**< 0.0001*** **	**< 0.0001*** **	**< 0.0001*** **	**< 0.0001*** **	**< 0.0001*** **
		η^2	0.75	0.51	0.68	0.29	0.51	0.68

WFE = wrist flexion-extension; WAA = wrist abduction-adduction; EPS = elbow pronation-supination; EFE = elbow flexion-extension. The critical value for Z is 1.96 (two-tailed). The adjusted alpha levels for the first to sixth most significant p values of the Wilcoxon signed-rank tests with Holm's sequential Bonferroni correction are 0.008, 0.010, 0.013, 0.017, 0.025 and 0.050 respectively. The levels of significant difference are reported at *P<Bonferroni adjusted alpha levels, **P<0.001 and ***P<0.0001.

b) Peak frequency of four tremor motions

Supplementary Table 6. Within-group comparison based on peak frequency.

Subject type	Action		EFE vs EPS	EPS vs WFE	EPS vs WAA	WAA vs WFE	EFE vs WFE	EFE vs WAA
PD (score < 0.50)	Resting (n = 54)	Z	0.0	-1.0	-0.9	0.0	-1.0	-1.2
		p	0.962	0.324	0.373	0.962	0.299	0.230
		η^2	<0.01	0.02	0.01	<0.01	0.02	0.03
	Outstretching (n = 46)	Z	-4.0	-1.3	-1.2	-2.2	-4.1	-2.0
		p	**<0.0001*** **	0.177	0.227	0.027	**<0.0001*** **	0.042
		η^2	0.35	0.04	0.03	0.11	0.37	0.09
	Wing (n = 49)	Z	-3.2	-1.3	-0.9	-0.1	-1.8	-2.4
		p	**0.001***	0.205	0.347	0.917	0.077	0.016
		η^2	0.21	0.03	0.02	<0.01	0.06	0.12
Normal	Resting (n = 62)	Z	-1.7	-2.7	-0.4	-2.1	-1.2	-1.2
		p	0.081	**0.007***	0.723	0.035	0.219	0.213
		η^2	0.05	0.12	<0.01	0.07	0.02	0.03
	Outstretching (n = 62)	Z	-5.2	-0.1	-1.3	-1.6	-5.1	-3.5
		p	**<0.0001*** **	0.958	0.198	0.102	**<0.0001*** **	**0.0004** **
		η^2	0.44	0.00	0.03	0.04	0.42	0.21
	Wing	Z	-2.4	-0.7	-0.3	-0.7	-2.8	-1.9

(n = 62)	*p*	0.015	0.460	0.782	0.508	**0.005***	0.063
	η^2	0.10	0.01	<0.01	0.01	0.13	0.06

WFE = wrist flexion-extension; WAA = wrist abduction-adduction; EPS = elbow pronation-supination; EFE = elbow flexion-extension. The critical value for Z is 1.96 (two-tailed). The adjusted alpha levels for the first to sixth most significant p values of the Wilcoxon signed-rank tests with Holm's sequential Bonferroni correction are 0.008, 0.010, 0.013, 0.017, 0.025 and 0.050 respectively. The significance of the difference is reported at *P<Bonferroni adjusted alpha levels, **P<0.001 and ***P<0.0001.

9. **Between-group comparison**

Supplementary Tables 7 and 8 show the statistical results of comparing subclinical PD and normal tremors based on the RMS $\Delta\theta_{joint}$ of individual motion and pair motions respectively. The statistical analysis results of the between-group comparison using peak frequency are tabulated in Supplementary Table 9. The explanation and discussions of the results are presented in the main manuscript.

a) Individual motion in RMS $\Delta\theta_{joint}$

Supplementary Table 7. Between-group comparison based on individual motion in RMS $\Delta\theta_{joint}$.

	WFE		WAA		EPS		EFE	
	χ2 (P value)	η2	χ2 (P value)	η2	χ2 (P value)	η2	χ2 (P value)	η2
Resting (54 PD vs 62 normal)	20.4<br>< 0.0001***	0.18	6.9<br>0.009*	0.06	21.5<br>< 0.0001***	0.19	17.1<br>< 0.0001***	0.15
Outstretching (46 PD vs 62 normal)	23.1<br>< 0.0001***	0.22	6.8<br>0.009*	0.06	17.4<br>< 0.0001***	0.16	12.1<br>0.0004**	0.11
Wing (49 PD 62 vs normal)	16.0<br>< 0.0001***	0.15	16.4<br>< 0.0001***	0.15	15.4<br>< 0.0001***	0.14	16.3<br>< 0.0001***	0.15

WFE = wrist flexion-extension; WAA = wrist abduction-adduction; EPS = elbow pronation-supination; EFE = elbow flexion-extension. The χ^2 (*p* values) are the results from Kruskal-Wallis. The critical χ^2 value for degrees of freedom, d.f. = 1 is 3.841 (one-tailed). The Eta-squared, η2 indicates the effect size. The significance of the difference is reported at * p < 0.05, ** p < 0.001 and *** p < 0.0001.

b) Pair motion in RMS $\Delta\theta_{joint}$

Supplementary Table 8. Between-group significant difference of the relative tremor severity between pair motions.

Subject type	Action		EPS – EFE	EPS – WFE	EPS –WAA
PD Score < 0.5 versus normal	Resting (54 PD vs 62 normal)	χ^2	10.2	3.5	15.7
		p	0.001**	0.060	< 0.0001***
		η^2	0.09	0.03	0.14
	Outstretching (46 PD vs 62 normal)	χ^2	1.3	2.4	10.2
		p	0.248	0.120	0.001**
		η^2	0.01	0.02	0.10
	Wing (49 PD vs 62 normal)	χ^2	3.5	2.6	5.7
		p	0.060	0.104	0.017*
		η^2	0.03	0.02	0.05

WFE = wrist flexion-extension; WAA = wrist abduction-adduction; EPS = elbow pronation-supination; EFE = elbow flexion-extension. The χ^2 (*p* values) are the results from Kruskal-Wallis. The critical χ^2 value for degrees of freedom, d.f. = 1 is 3.841 (one-tailed). The Eta-squared, η2 indicates the effect size. The significance of the difference is reported at * $p < 0.05$, ** $p < 0.001$ and *** $p < 0.0001$.

c) Individual motion in peak frequency

Supplementary Table 9. Significant difference between the peak frequency of PD and normal subject tremors in different motions.

	WFE		WAA		EPS		EFE	
	$\chi 2$ (P value)	$\eta 2$	$\chi 2$ (P value)	$\eta 2$	$\chi 2$ (P value)	$\eta 2$	$\chi 2$ (P value)	$\eta 2$
Resting (54 PD vs 62 normal)	1.6 (0.21)	0.01	1.0 (0.31)	0.01	0.5 (0.47)	<0.01	0.5 (0.50)	<0.01
Outstretching (46 PD vs 62 normal)	5.6 (0.02)*	0.05	2.2 (0.14)	0.02	2.2 (0.14)	0.02	11.7 (0.0006)**	0.11
Wing (49 PD vs 62 normal)	<0.1 (0.84)	<0.01	0.7 (0.40)	<0.01	0.5 (0.48)	<0.01	<0.1 (0.92)	<0.01

WFE = wrist flexion-extension; WAA = wrist abduction-adduction; EPS = elbow pronation-supination; EFE = elbow flexion-extension. The χ^2 (*p* values) are the results from Kruskal-Wallis. The critical χ^2 value for degrees of freedom, d.f. = 1 is 3.841 (one-tailed). The Eta-squared, $\eta 2$ indicates the effect size. The significance of the difference is reported at * $p < 0.05$, ** $p < 0.001$ and *** $p < 0.0001$.

10. Summary of the tremor motion characteristics

Though all the tremors had no clinical sign, the characteristics that are unique to PD and normal tremors are found in the analysis (see Supplementary Table 10). In between-group comparison especially, PD tremors are significantly different from normal tremors. The median values of the rest and postural conditions of baseline and PD tremors having no clinical sign based on predicted rating are reported for the first time.

The severity ranking of the PD and normal tremors in increasing order is WAA, WFE, EFE, and EPS. This order is found in all actions except for the WFE and EFE in wing posture of PD tremors and the wrist motions in rest condition of normal tremor. The rank order indicates that the tremor about the elbow has larger amplitude compared to tremor about the wrist joint in most of the tremor cases.

PD and normal tremor share the common characteristics of having EPS as the most predominant tremor motion and WAA as the least predominant tremor motion. Nevertheless, such predominance in PD tremor is significantly greater than that of normal tremor, as supported by a significantly larger EPS–WAA value in the former tremor. This suggests that the relative severity of the two motions in PD tremor is not a mere manifestation of physiological tremor.

Peak frequencies of all three actions are not significantly distinguishable by all tremor motions except for EFE and WFE in outstretching posture that PD tremor is of higher peak frequency compared to normal tremor. This makes EFE and WFE in outstretching posture the only motions that give distinct difference between PD and normal tremors based on both amplitude and frequency. The findings of rank order and distinct peak frequency of PD tremor in specific motion are new.

Supplementary Table 10. Summary of the tremor motion characteristics of subclinical PD and normal tremors

Within-group comparison			
Parameter	Posture/action	Subclinical PD	Normal
RMS $\Delta\theta_{joint}$	Rest	WAA < WFE	No significant difference between WFE and WAA
	Wing	No significant difference between EFE and WFE	WFE < EFE
Peak frequency	Rest	No significant difference in all comparisons	WFE < EPS
	Outstretched	EFE < EPS and WFE	EFE < EPS, WFE and WAA

	wing	EFE < EPS	EFE < WFE

PD tremor versus normal tremor

Parameter	Posture/action	Motion-related parameters with significant difference
RMS $\Delta\theta_{joint}$	Rest	EPS – EFE (pair-motion difference)
	Rest, outstretched and wing	EPS – WAA (pair-motion difference)
Peak frequency	Outstretched	EFE, WFE (individual motion)

11. Tremors involving patients with medication intake of within and greater than 3 h

Statistical analysis was performed to compare the difference between PD with medication intake of within three hours (subgroup I) and another subgroup with medication intake of within three hours removed (subgroup Ii). The results are as tabulated in Supplementary Table 11. A further analysis was carried out to compare the characteristics of normal and PD subject groups by excluding the patients with < 3 h of medication intake. The results are as shown in Supplementary Table 12. Kruskal-Wallis tests were performed for both statistical analyses. Supplementary Tables 13 and 14 are the median of the individual and pair motion difference in subgroup II. Supplementary Figure 3 shows the boxplots of the RMS$\Delta\theta_{joint}$ of pair motions for subgroups I and II for different actions. The median values of the individual and pair motion difference in subgroup II are provided in the Supplementary Tables 13 and 14 respectively.

Supplementary Table 11. Tremor characteristics significant difference between PD subgroup with medication intake < 3 h and PD subgroup with medication intake for ≥ 3 h.

Action		WFE	WAA	EPS	EFE	EPS – EFE	EPS – WFE	EPS –WAA
Resting (43 subgroup II vs 11 subgroup I)	χ^2	0.033	0.084	0.288	0.684	0.234	0.175	0.175
	p	0.855	0.772	0.591	0.408	0.629	0.675	0.675
	η^2	<0.01	<0.01	0.01	0.01	<0.01	<0.01	<0.01
Outstretching (38 subgroup II vs 8 subgroup I)	χ^2	2.271	0.001	1.150	0.142	0.971	1.277	0.121
	p	0.132	0.977	0.284	0.706	0.324	0.258	0.728
	η^2	0.05	<0.01	0.02	<0.01	<0.01	0.02	0.03
Wing (38 subgroup II vs 11 subgroup I)	χ^2	0.625	0.069	0.230	0.450	0.129	0.873	1.268
	p	0.429	0.792	0.632	0.502	0.719	0.350	0.260
	η^2	0.01	<0.01	<0.01	0.01	0.03	<0.01	0.02

WFE = wrist flexion-extension; WAA = wrist abduction-adduction; EPS = elbow pronation-supination; EFE = elbow flexion-extension. The χ^2 (*p* values) are the results from Kruskal-Wallis. The critical χ^2 value for degrees of freedom, d.f. = 1 is 3.841 (one-tailed). The Eta-squared, η2 indicates the effect size. The significance of the difference is reported at * $p < 0.05$, ** $p < 0.001$ and *** $p < 0.0001$. The terms "subgroup II" and "subgroup I" refer to the PD subgroups without and with the last dose of medication taken within 3 h before the measurement. $RMS\Delta\theta_{joint}$ was used to quantify the tremor severity.

Supplementary Table 12. Between-group significant difference of the relative tremor severity between pair motions.

Subject type	Action		EPS – EFE	EPS – WFE	EPS – WAA
PD Score < 0.5 versus normal	Resting (43 subgroup II vs 62 normal)	χ^2	7.1	2.2	14.8
		p	0.003*	0.056	<0.001**
		η^2	0.08	0.04	0.14
	Outstretching (38 subgroup II vs 62 normal)	χ^2	1.5	0.7	8.3
		p	0.297	0.057	0.010*
		η^2	0.01	0.04	0.07
	Wing (38 subgroup II vs 62 normal)	χ^2	1.5	1.5	4.2

p	0.219	0.191	0.079
η^2	0.02	0.02	0.03

WFE = wrist flexion-extension; WAA = wrist abduction-adduction; EPS = elbow pronation-supination; EFE = elbow flexion-extension. The χ^2 (p values) are the results from Kruskal-Wallis. The critical χ^2 value for degrees of freedom, d.f. = 1 is 3.841 (one-tailed). The Eta-squared, η^2 indicates the effect size. The significance of the difference is reported at * p < 0.05, ** p < 0.001 and *** p < 0.0001. The terms "subgroup II" and "subgroup I" refer to the PD subgroups without and with the last dose of medication taken within 3 h before the measurement. $RMS\Delta\theta_{joint}$ was used to quantify the tremor severity.

Supplementary Table 13. Median of the individual tremor motions in PD subgroup II

	Action	EPS	EFE	WFE	WAA
$RMS\Delta\theta_{joint}$	Resting (n = 43)	0.019 (0.016, 0.022)	0.012 (0.010, 0.013)	0.009 (0.008, 0.011)	0.008 (0.007, 0.010)
	Outstretching (n = 38)	0.030 (0.024, 0.034)	0.018 (0.016, 0.025)	0.014 (0.012, 0.016)	0.010 (0.009, 0.013)
	Wing (n = 38)	0.024 (0.021, 0.029)	0.017 (0.015, 0.019)	0.014 (0.010, 0.016)	0.011 (0.008, 0.014)
Peak frequency	Resting (n = 43)	4.9 (4.3, 5.3)	5.0 (4.5, 5.5)	5.0 (4.3, 5.4)	4.7 (4.3, 5.3)
	Outstretching (n = 38)	5.8 (4.9, 7.0)	4.9 (4.2, 5.3)	7.0 (5.8, 8.1)	5.3 (4.3, 7.1)
	Wing (n = 38)	4.4 (4.1, 4.9)	3.9 (3.7, 4.4)	4.1 (3.8, 4.8)	4.1 (3.8, 4.9)

The median values are in ˚. n = number of sample.

Supplementary Table 14. Median of the pair motions in PD subgroup I

	Action	EPS – EFE	EPS – WFE	EPS – WAA
$RMS\Delta\theta_{joint}$	Resting (n = 43)	0.008 (0.006, 0.011)	0.009 (0.007, 0.011)	0.010 (0.008, 0.013)
	Outstretching (n = 38)	0.010 (0.006, 0.018)	0.015 (0.011, 0.021)	0.018 (0.014, 0.022)

Wing (n = 38)	0.009 (0.007, 0.014)	0.012 (0.010, 0.020)	0.013 (0.012, 0.019)

The median values are in ˚. n = number of sample.

(A)

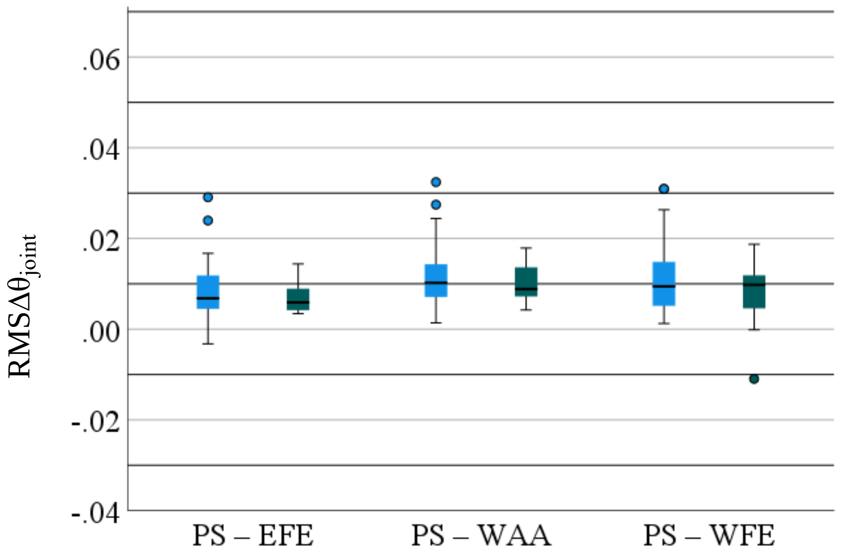

(B)

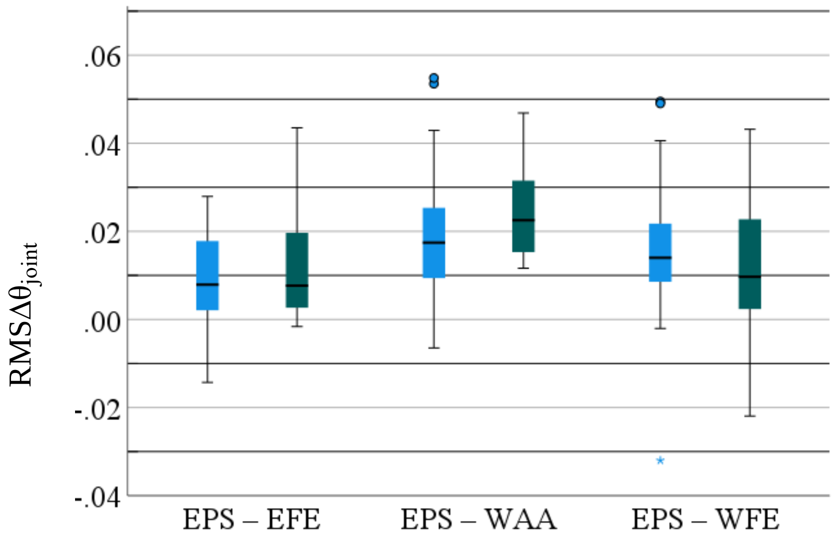

(C)

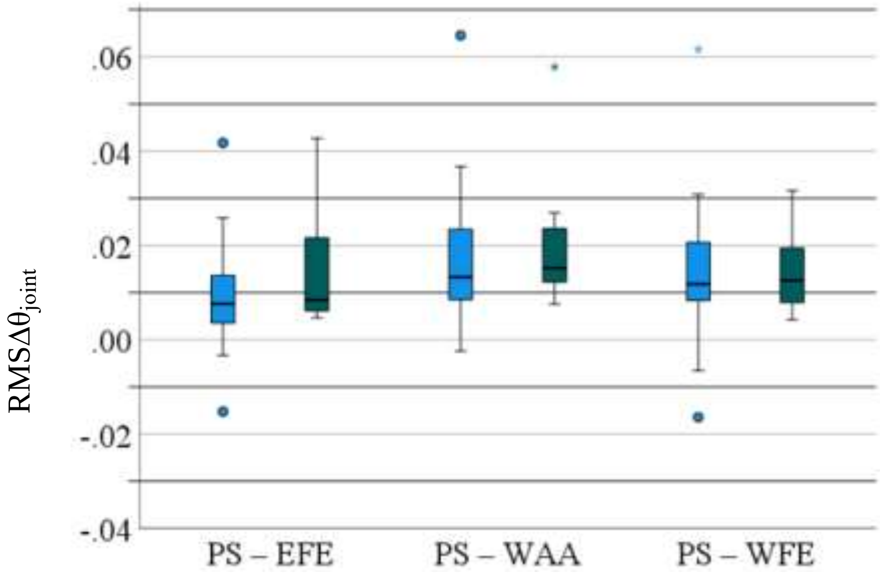

Supplementary Figure 3. RMS$\Delta\theta_{joint}$ of pair motions for subgroups I and II during (A) rest, (B) outstretching and (C) wing actions. The terms "subgroup II" and "subgroup I" refer to the PD subgroups without and with the last dose of medication taken within 3 h before the measurement.

References

1 Van Den Eeden, S. K. *et al.* Incidence of Parkinson's disease: variation by age, gender, and race/ethnicity. *Am J Epidemiol* **157**, 1015-1022 (2003).
2 Hirsch, L., Jette, N., Frolkis, A., Steeves, T. & Pringsheim, T. The incidence of Parkinson's disease: a systematic review and meta-analysis. *Neuroepidemiology* **46**, 292-300 (2016).
3 Tomlinson, C. L. *et al.* Systematic review of levodopa dose equivalency reporting in Parkinson's disease. *Movement disorders* **25**, 2649-2653 (2010).
4 Cohen, J. *Statistical power analysis for the behavioral sciences*. 2nd edn, (L. Erlbaum Associates, 1988).
5 Sternberg, E. J., Alcalay, R. N., Levy, O. A. & Louis, E. D. Postural and intention tremors: a detailed clinical study of essential tremor vs. Parkinson's disease. *Frontiers in neurology* **4** (2013).
